# Comparison of Advanced Dynamic Arc Therapy With Collimator Rotation and Fixed Integrated Gantry Positions to the Standard of Care Across Five Treatment Sites

**DOI:** 10.7759/cureus.86280

**Published:** 2025-06-18

**Authors:** Ryan Clark, Anthony Magliari, Lesley Rosa, Taoran Li, Sushil Beriwal, Luca Cozzi

**Affiliations:** 1 Department of Medical Affairs, Varian, Palo Alto, USA

**Keywords:** dosimetric scorecard, plan quality, rapidarc dynamic, treatment planning, vmat

## Abstract

Aim

To evaluate dosimetric plan quality, optimization efficiency, and delivery time of RapidArc Dynamic (RAD), a novel treatment planning approach combining dynamic gantry positions with dynamic collimator rotation and user-selected static angle modulated ports (STAMPs), compared to conventional volumetric modulated arc therapy (VMAT) across five anatomical treatment sites.

Methods

Treatment plans were developed for breast, lung, pancreas/liver, prostate, and head and neck cases. VMAT plans were first optimized following clinical guidelines and dosimetric scorecards. The same optimization objectives from the VMAT plans were applied to the RAD plans. RAD-specific variables, including STAMP selection and weighting, were systematically explored. When the best settings for RAD were found, optimization objectives were modified to further improve quality specific to each case. For equal comparison between the two techniques, the optimization objectives from the RAD plans were copied back to the VMAT plans for re-optimization. Optimization time, monitor units, number of control points, delivery time, and dosimetric plan quality were then compared.

Results

RAD demonstrated reduced optimization times compared to VMAT, averaging one minute and 23 seconds versus two minutes and seven seconds. Dosimetric improvements were observed across all sites: breast plans showed reduced heart mean dose (1.98 vs. 1.23 Gy) and improved dose homogeneity; lung plans achieved better heart V5Gy; prostate plans demonstrated enhanced bladder and rectum sparing at V18Gy; and head and neck plans showed improved sparing of the lips and oral cavity. Treatment delivery times were reduced in most cases, with improvements ranging from 11% to 25% in breast, lung, and head and neck.

Conclusions

RAD offers faster optimization times while maintaining or improving plan quality compared to VMAT. The integration of dynamic collimator rotation and strategic STAMPs can provide enhanced organ-at-risk sparing without compromising target coverage. These improvements, combined with generally reduced delivery times, suggest RAD offers a potential advancement in radiation therapy planning efficiency and plan quality.

## Introduction

Volumetric modulated arc therapy (VMAT) is one of the commonly used techniques in radiation therapy, having evolved from intensity-modulated radiation therapy (IMRT). VMAT revolutionized treatment delivery by allowing simultaneous variation of gantry rotation speed, treatment aperture shape, and dose rate. This technique offered advantages over conventional IMRT, including shorter treatment times, reduced monitor units (MU), and the potential for improved dose distributions. Advancements in linear accelerator technology and treatment planning systems facilitated VMAT’s quick adoption, establishing it as a standard of care in modern radiation oncology for treating various cancer types [1].

Published literature suggests that the combination of VMAT and fixed beams can increase plan quality by improving organ at risk (OAR) doses in certain clinical scenarios [2,3]. Matuszak et al. introduced FusionArc optimization, which combined IMRT and VMAT, resulting in an excellent balance of treatment plan quality and efficiency [4]. A similar approach was also validated by Akbas et al., who demonstrated significant improvements in target dose homogeneity and conformity compared to standalone IMRT and VMAT techniques [5]. For breast cancer patients, Lin et al. reported superior heart sparing with hybrid techniques while maintaining adequate target coverage [6]. More recently, Raturi et al. demonstrated that hybrid IMRT/VMAT significantly improved target conformity for olfactory neuroblastoma while providing better sparing of the contralateral optic nerve compared to both non-coplanar VMAT and IMRT approaches [7]. However, the current process of optimizing plans with a hybrid approach can be complicated and time-consuming.

RapidArc Dynamic (RAD), available in Varian Eclipse V18.1, builds upon VMAT to offer enhanced treatment capabilities. This advanced solution integrates the following: dynamic gantry rotation; pausing gantry position; dynamically rotating collimation in a single (or multiple) treatment arc(s) with contiguous multi-leaf collimator (MLC) apertures. Enabling this capability can increase the number of machine control points (CPs) used during treatment. RAD employs an all-new optimizer with efficiently generated MLC sequencing in a pure graphic processing unit (GPU) implementation, which accelerates treatment planning optimization. The system allows for greater modulation from key user-selected static angle modulated ports (STAMPs). We predicted that RAD’s integration of dynamic collimator rotation with strategically placed STAMPs would achieve improved or equivalent dosimetric plan quality compared to conventional VMAT while reducing optimization times and potentially decreasing treatment delivery times across different anatomical sites. The specific objectives of this study were to evaluate the dosimetric plan quality of RAD compared to conventional VMAT across five diverse anatomical treatment sites, assess optimization efficiency differences between RAD and VMAT techniques, measure treatment delivery time differences between the two approaches, and determine optimal RAD configuration parameters (STAMP selection, weighting, and arc geometry) for each anatomical site.

## Materials and methods

Study overview

This study examined the potential of RAD across five diverse cancer case types: breast with simultaneous integrated boost (SIB), locally advanced lung, pancreas/liver, prostate, and head and neck. One representative case was selected for each of the five anatomical sites, with multiple optimization iterations performed to determine the optimal RAD configuration. VMAT plans were optimized using two full arc rotations (except four arcs for the head and neck case) from 181° to 179° clockwise and counterclockwise with a fixed collimator to establish baseline plan quality according to clinical guidelines and dose metrics [8-12]. These optimization objectives were then applied to RAD plans, which underwent an iterative process to assess how plan quality and optimization cost functions changed with new configurable options now available with RAD.

Convergence mode and iteration settings

The same GPU hardware (NVIDIA L4 Tensor Core) was used for all optimizations. For VMAT, all three optimization convergence modes were compared: off (default), on, and extended (maximum allowable). The convergence mode setting controls how thoroughly the optimizer searches for optimal solutions that best match the optimization objectives, with “off” representing the fastest optimization, “on” recommended for daily clinical use, providing improved plan quality, and “extended” offering the most thorough optimization at the cost of longer computation times. Similarly, for the photon optimizer used in RAD, iterations of 800 (default), 2000, and 4000 (maximum allowable) were evaluated. These comparisons were made for each treatment site, analyzing the effects of optimization time on plan MU and dosimetric plan scores.

VMAT beam configuration

VMAT plans employed standard clinical beam arrangements with specific arc configurations optimized for each anatomical site. The breast SIB case utilized two arcs spanning 168-304° (clockwise/counterclockwise) with 224 total CPs and fixed collimator angles. The lung case used two arcs from 181° to 19.5° (clockwise/counterclockwise) with a total of 200 CPs. Pancreas/liver cases employed two arcs from 181° to 90° (clockwise/counterclockwise) with a total of 268 CPs. Prostate cases used two full arcs from 181° to 179° (clockwise/counterclockwise) with a total of 360 CPs. Head and neck cases utilized four arcs from 181° to 179° (clockwise/counterclockwise) with 720 total CPs. All VMAT plans maintained fixed collimator angles throughout arc delivery (Table 1).

**Table 1:** Beam delivery parameters for VMAT versus RAD across five anatomical sites ^*^ CPs indicate plan modulation complexity. ^†^ STAMP positions refer to user-selected gantry pause angles. Static weighting reflects the balance between arc delivery and static beam contribution, where 0 denotes a balanced distribution and +1 indicates static beam weighting. RAD demonstrated enhanced modulation for complex cases, showing increases of 56% for pancreas/liver and 12% for prostate, while improving efficiency in other sites, with reductions of 5% for breast and 3% for head and neck, all while using fewer arcs. CCW, counterclockwise; CP, control point; CW, clockwise; PO, photon optimizer; RAD, RapidArc Dynamic; SIB, simultaneous integrated boost; STAMP, static angle modulated port; VMAT, volumetric modulated arc therapy

Treatment site	VMAT arcs	VMAT arc range	VMAT CPs^*^	RAD arcs	RAD STAMPs	RAD STAMP angles	STAMP weighting^†^	RAD CPs	Modulation comparison
Breast SIB	2	168-304° (CW/CCW)	224	1	4	304°, 320°, 128°, 144°	+0 (balanced)	213	-5% CPs, fewer arcs
Lung	2	181-19.5° (CW/CCW)	200	1	4	189°, 197°, 5°, 13°	+0 (balanced)	200	Same CPs, fewer arcs
Pancreas/liver	2	181-90° (CW/CCW)	268	2	3+3	Arc 1: 186°, 308°, 345°; Arc 2: 285°, 335°, 9°	0 (balanced)	417	+56% CPs, same arcs
Prostate	2	181-179° (CW/CCW)	360	1	6	212.7°, 245.5°, 311°, 49.3°, 114.9°, 147.6°	+1 (static)	404	+12% CPs, fewer arcs
Head and neck	4	181-179° (CW/CCW)	720	2	6+1	Arc 1: 311°, 343.8°, 16.6°, 49.3°, 114.9°, 149°; Arc 2: 0°	+1 (static)	697	-3% CPs, fewer arcs

RAD-specific planning variables

A full exploration of STAMP weighting adjustments, “arc dominant” (-2), “arc” (-1), “balanced” (0), “static” (+1), and “static dominant” (+2), was tested for each plan. The number of additional MLC CPs utilized in the STAMP varies from only two additional CPs for each static gantry position when arc dominant is selected to a maximum of 51 on the other extreme when static dominant is used. In all cases, except the pancreas/liver, to maintain dose conformity, the number of arcs was halved as well. All RAD plans employed fully optimized, automatic dynamic collimator rotation (except the second arc of the head and neck case, where the collimator was fixed at 90° to maintain lateral coverage where the PTV was widest and reduce MLC travel due to the target size). Exploring these planning variables was performed while keeping optimization objectives, structure contours, and dose calculation settings constant.

The user-defined STAMPs (or gantry pause positions) for each treatment site were tested at various key gantry angles. Those possible static angle selections were narrowed by avoiding beam trajectories that pass directly through critical OARs and by identifying regions where VMAT plans exhibited higher dose rates/slower gantry rotation speeds as CPs that could potentially benefit from additional modulation. The specific STAMP configurations used for each anatomical site are as follows: breast SIB used four STAMPs at 304°, 320°, 128°, and 144° with balanced weighting; lung used four STAMPs at 189°, 197°, 5°, and 13° with balanced weighting; pancreas/liver used 3 + 3 STAMPs across two arcs (Arc 1: 186°, 308°, and 345°; Arc 2: 285°, 335°, and 9°) with balanced weighting; prostate used six STAMPs at 212.7°, 245.5°, 311°, 49.3°, 114.9°, and 147.6° with static weighting (+1); and head and neck used 6 + 1 STAMPs across two arcs (Arc 1: 311°, 343.8°, 16.6°, 49.3°, 114.9°, and 149°; Arc 2: 0°) with static weighting (+1) (Table 1).

Automatic skin flash

RAD incorporates an automated skin flash management function that addresses the clinical need for adequate dose coverage in free-breathing breast treatments while minimizing planning complexity. The algorithm expands the target structure in six directions to form a virtual target with corresponding relative optimization objectives, creates a skin flash target from the original target by a user-defined distance, and generates a virtual bolus structure by subtracting the body contour from the skin flash target to create a variable density region outside the patient surface. Angle-dependent density is applied with density increasing toward user-defined density as gantry angles become more tangent to the skin surface. As a result, when the gantry approaches en face, an air equivalent density is used. Users can customize skin flash extension distance and bolus densities to meet specific clinical requirements (-300 HU and 2.0 cm used in this work). This approach eliminated the more time-intensive manual bolus method required for the VMAT technique and provided improved dose homogeneity for the RAD breast case.

Finalizing optimization objectives for both RAD and VMAT

After setting the RAD beam configuration, objectives were fine-tuned to maximize OAR sparing while maintaining target coverage and dose heterogeneity for RAD plans. This fine-tuning involved adjusting the priorities, dose values, or volume constraints of the optimization objectives, allowing for more aggressive OAR sparing or improved target coverage. A systematic approach to adjusting the optimization objectives was used, where dose values were decreased first, followed by volume constraints in relative increments of one to three percent. The priority of these objectives would then be increased, relative to the target objectives, if the cost function were less than two percent. Finally, these new RAD plan objectives were applied back to the original VMAT plans with two arcs (four arcs for the head and neck case) for fair evaluation of RAD’s performance relative to current VMAT techniques. All plans were created and reviewed by the same treatment planner with consistent optimization methodology to reduce plan variability.

Plan quality assessment with clinical goals and dosimetric scorecards

Optimization objective tuning results were evaluated with plans normalized to match the target coverage of the lowest dose target (when applicable) using clinical goals and dosimetric scorecards. Dosimetric scorecards add additional, often aspirational, dose-volume histogram points to existing metrics in piecewise linear scoring functions for each metric, which are summed to a total score [13-17]. These scorecards include several additional metrics that award points for target homogeneity and the conformity of multiple isodose levels, among others. Complete scorecards are included with the full DICOM export of cases online [18] along with the scoring tool [19]. Beyond dosimetric evaluation, total MU and treatment delivery time were also measured on a TrueBeam v4.1 with field automation enabled, timed from first beam on to last beam off.

## Results

Convergence mode and iteration analysis

The impact of different convergence modes (VMAT) and number of iterations (RAD) on plan quality and MUs was evaluated (Table 2). This analysis aimed to provide insights into the relative performance and efficiency of RAD compared to traditional VMAT techniques across various convergence settings, where both VMAT and RAD demonstrate increasing performance with further optimization, but VMAT requires substantially more time to achieve the highest dosimetric quality.

**Table 2:** Plan comparison summary between PO and RAD for five cases with different convergence modes RAD plans are labeled with their respective STAMP weighting, number of arcs, and angles in each title by case. MU, monitor unit; PO, photon optimizer; RAD, RapidArc Dynamic; STAMP, static angle modulated port

Convergence mode comparison						
Breast (out of 141 points)						
Plan type	PO			RAD +0_1arc_4Static		
Convergence mode	Off	On	Extended	800	2000	4000
Optimization time (seconds)	101	229	722	24	42	73
Plan MU	842	833	913	946	1000	1036
Plan score	104.93	117.17	120.6	124.95	126.91	128.4
Lung (out of 156 points)						
Plan type	PO			RAD +0_1arc_4Static		
Convergence mode	Off	On	Extended	800	2000	4000
Optimization time (seconds)	95	210	980	20	34	55
Plan MU	721	741	784	749	744	742
Plan score	95.45	99.77	105.28	104.07	106.22	107.26
Pancreas/liver (out of 150 points)						
Plan type	PO			RAD 0_2arc_3+3Static		
Convergence mode	Off	On	Extended	800	2000	4000
Optimization time (seconds)	142	319	1043	24	45	73
Plan MU	3535	3847	4344	3630	3575	3630
Plan score	134.16	139.74	139.24	139.99	139.96	139.66
Prostate (out of 200 points)						
Plan type	PO			RAD +1_1arc_6Static		
Convergence mode	Off	On	Extended	800	2000	4000
Optimization time (seconds)	156	310	1431	34	50	76
Plan MU	2231	2293	2328	3588	3605	3602
Plan score	167.87	172.6	178.29	177.41	177.39	178.02
Head and neck (out of 240 points)						
Plan type	PO (4arc)			RAD +1_2arcs_8+1Static		
Convergence mode	Off	On	Extended	800	2000	4000
Optimization time (seconds)	144	339	1206	53	83	136
Plan MU	924	957	988	1186	1182	1182
Plan score	181.45	189.85	194.41	189.7	194.03	196.45

**Figure 1:**
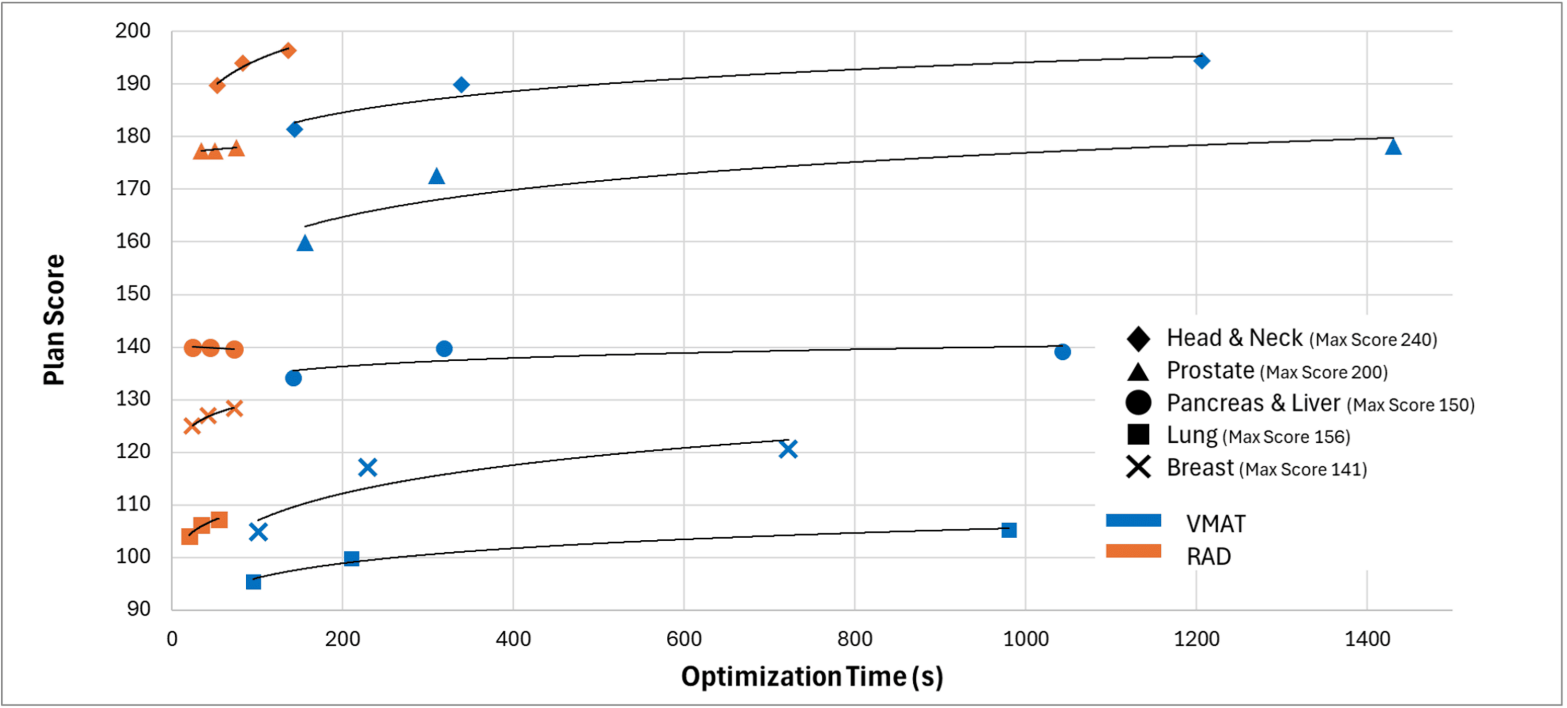
Optimization time versus plan score for each treatment site using VMAT and RAD Plan scores increase with optimization time, indicating a trade-off between plan quality and efficiency. Solid line added to better differentiate treatment sites and show trend, not to be interpreted as regression, with color differentiation between VMAT (blue) and RAD (orange). RAD, RapidArc Dynamic; VMAT, volumetric modulated arc therapy

Table 2 demonstrates that while VMAT’s extended convergence mode can achieve higher plan quality scores, the increased optimization times make it less practical for routine clinical workflows. RAD consistently achieved improved plan quality compared to VMAT’s fastest convergence setting (off) across all anatomical sites. When VMAT is allowed maximum optimization time with extended convergence mode, RAD’s efficiency advantage becomes more pronounced, reaching superior plan quality in approximately one to two minutes compared to VMAT extended mode, requiring 12 to 24 minutes. Figure 1 illustrates the complete optimization landscape across all available settings for both techniques, showing that while VMAT Extended mode can achieve higher plan quality, optimization times increase by 700-1400 seconds. 

Feature comparison plan results

**Figure 2:**
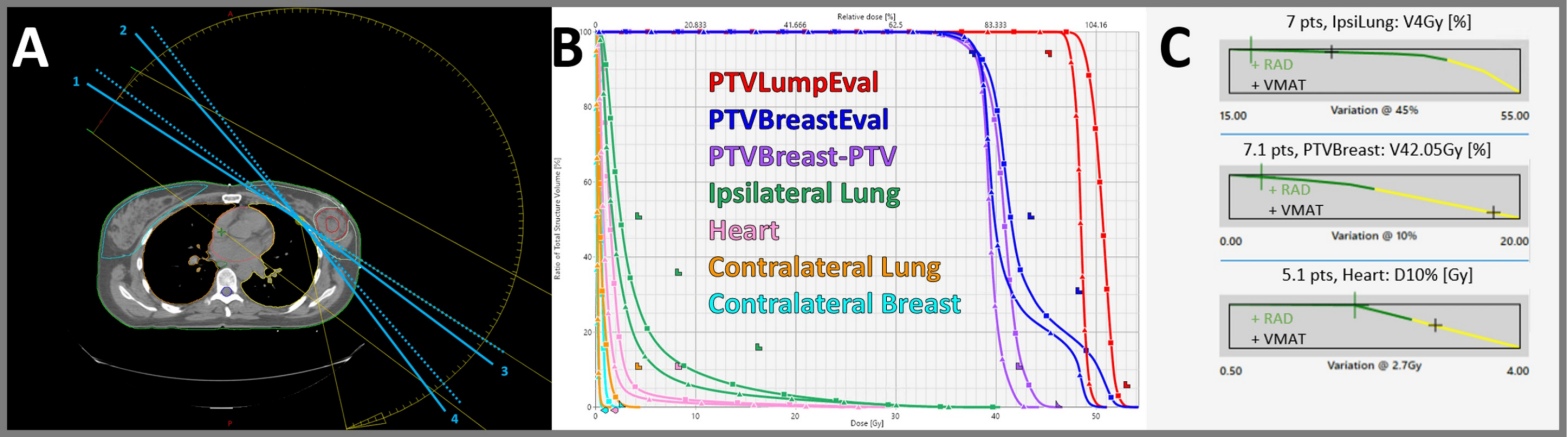
SIB breast (48/40 Gy in 15 fractions) (A) Arc trajectory with four STAMP selections (solid line entrance and dotted line exit). (B) DVH comparison for VMAT (squares) and RAD (triangles). (C) Example scorecard metric piecewise linear functions for VMAT and RAD. DVH, dose-volume histogram; RAD, RapidArc Dynamic; SIB, simultaneous integrated boost; STAMP, static angle modulated port; VMAT, volumetric modulated arc therapy

**Figure 3:**
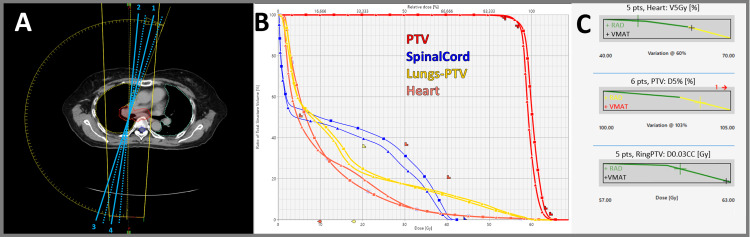
Lung (60 Gy in 30 fractions) (A) Arc trajectory with four STAMP selections (solid line entrance and dotted line exit). (B) DVH comparison for VMAT (squares) and RAD (triangles). (C) Example scorecard metric piecewise linear functions for VMAT and RAD. DVH, dose-volume histogram; RAD, RapidArc Dynamic; STAMP, static angle modulated port; VMAT, volumetric modulated arc therapy

**Figure 4:**
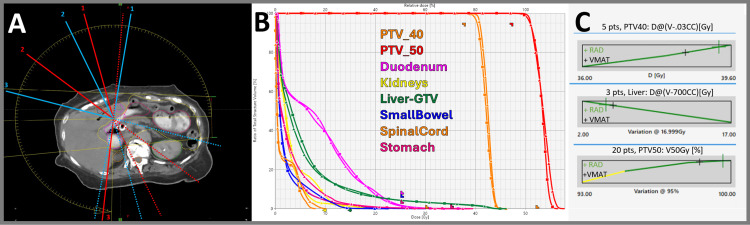
Pancreas/liver (50/40 Gy in five fractions) (A) Arc trajectory (two arcs) with three STAMP selections per arc (blue labeled as the first arc and red as the second arc, dotted lines exit). (B) DVH comparison for VMAT (squares) and RAD (triangles). (C) Example scorecard metric piecewise linear functions for VMAT and RAD. DVH, dose-volume histogram; RAD, RapidArc Dynamic; STAMP, static angle modulated port; VMAT, volumetric modulated arc therapy

**Figure 5:**
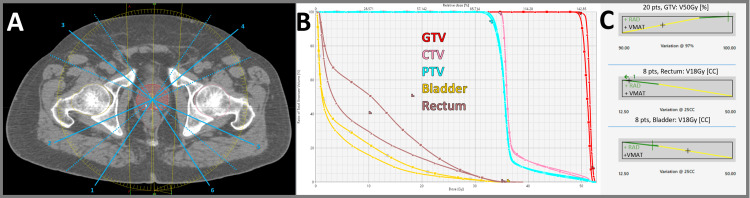
SBRT prostate (50/35 Gy in five fractions) (A) Arc trajectory with six STAMP selections (solid line entrance and dotted line exit). (B) DVH comparison for VMAT (squares) and RAD (triangles). (C) Example scorecard metric piecewise linear functions for VMAT and RAD. DVH, dose-volume histogram; RAD, RapidArc Dynamic; SBRT, stereotactic body radiation therapy; STAMP, static angle modulated port; VMAT, volumetric modulated arc therapy

**Figure 6:**
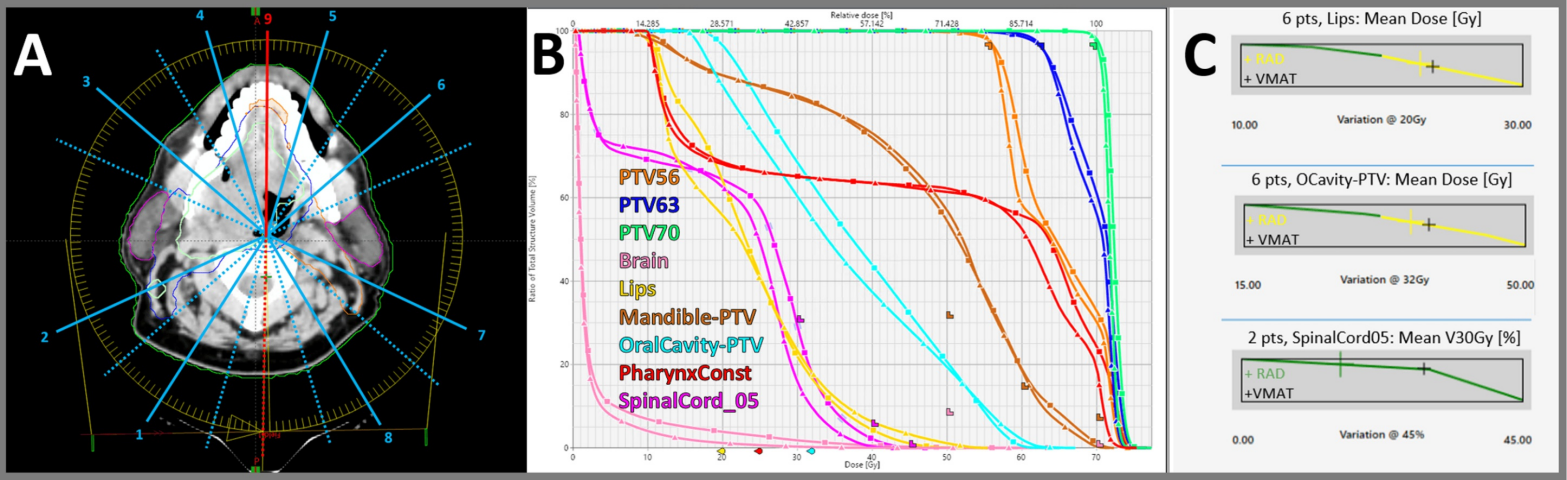
Head and neck (70, 63, and 56 Gy in 35 fractions) (a) Two arc trajectories with nine total STAMPs (blue labeled as the first arc and red as the second arc, dotted lines exit). (b) DVH comparison for VMAT (squares) and RAD (triangles). (c) Example scorecard metric piecewise linear functions for VMAT and RAD. DVH, dose-volume histogram; RAD, RapidArc Dynamic; STAMP, static angle modulated port; VMAT, volumetric modulated arc therapy

While all data is presented for each case, this study directly compares RAD (4000 iterations) to VMAT (convergence mode = off) as optimization times are most similar across five different case types: breast with SIB (Figure 2), lung (Figure 3), pancreas/liver (Figure 4), prostate (Figure 5), and head and neck (Figure 6). Plan optimization time, plan MU, dosimetric quality plan score, and plan delivery time were evaluated.

In RAD’s highest quality setting (4000 iterations), it demonstrated reduced optimization times compared to VMAT’s fastest setting (convergence mode = off). RAD average optimization time was one minute and 23 seconds compared to VMAT, two minutes and seven seconds, representing a 35% reduction.

MU and delivery time

In terms of treatment time and total MU, RAD plans showed an increase in MU while delivery times were on average lower, by seven seconds (Table 3), with the breast, lung, and head and neck cases demonstrating reductions of 11-25%. RAD plans are typically delivered with a higher average dose rate due to simpler MLC shapes and transitions, resulting in fewer drops in dose rate. RAD reduced delivery times compared to VMAT for breast (82 seconds vs. 93 seconds), lung (64 seconds vs. 85 seconds), and head and neck (282 seconds vs. 315 seconds). The prostate case demonstrated similar treatment delivery times (146 seconds vs. 145 seconds) even though RAD increased MU (+61.5%). RAD delivery times increased in the pancreas/liver case (202 seconds vs. 166 seconds), where both arcs from VMAT were included to enhance target dose conformality and prevent dose bridging for multiple targets.

**Table 3:** Plan delivery time (measured from first beam on through final beam off) on phantom with field automation enabled between VMAT (convergence mode = off) and RAD (iterations = 4000) for five cases MU, monitor unit; RAD, RapidArc Dynamic; VMAT, volumetric modulated arc therapy

Delivered plan comparison summary										
Treatment site	Breast		Lung		Pancreas/liver		Prostate		Head and neck	
Plan type	VMAT	RAD	VMAT	RAD	VMAT	RAD	VMAT	RAD	VMAT	RAD
Optimization time (seconds)	101	73	95	55	142	73	156	76	144	136
Plan MU	842	1036	721	742	3535	3630	2231	3602	924	1182
Plan score	105	128	95	107	134	140	168	178	181	196
Plan delivery time (seconds)	93	82	85	64	166	202	145	146	315	282

Dosimetric quality by treatment site

Clinical goals were met by both techniques on both the pancreas/liver and head and neck, whereas more goals were met with RAD on the remaining three cases (Table 4, Table 5, Table 6, Table 7, Table 8).

**Table 4:** Comparison of dose metrics between VMAT and RAD for the SIB breast case SIB, simultaneous integrated boost; RAD, RapidArc Dynamic; VMAT, volumetric modulated arc therapy

SIB breast clinical goals				
Structure	Priority	Metric	VMAT	RAD
PTVLumpEval	P1	Dmax ≤ 55.00 Gy	54.25 Gy	51.14 Gy
PTVLumpEval	P1	V 52.80 Gy ≤ 5.0%	0.57%	0.00%
PTVLumpEval	P1	V 45.60 Gy ≥ 95.0%	100.00%	100.00%
CONTRA_BREAST	P2	Dmean ≤ 1.00 Gy	0.61 Gy	0.15 Gy
CONTRA_BREAST	P2	Dmax ≤ 2.40 Gy	2.51 Gy	0.74 Gy
CONTRA_LUNG	P2	V 4.00 Gy ≤ 10.0%	0.01%	0.00%
HEART	P2	V 8.00 Gy ≤ 10.0%	2.28%	1.35%
HEART	P2	Dmean ≤ 2.00 Gy	1.98 Gy	1.23 Gy
IPSILATERAL_LUNG	P2	V 4.00 Gy ≤ 50.0%	29.13%	18.03%
IPSILATERAL_LUNG	P2	V 8.00 Gy ≤ 35.0%	12.36%	7.28%
IPSILATERAL_LUNG	P2	V 16.00 Gy ≤ 15.0%	4.65%	2.99%
PTV Breast – PTV	P1	Dmax ≤ 46.00 Gy	46.70 Gy	44.35 Gy
PTV Breast – PTV	P1	V 42.00 Gy ≤ 10.0%	18.93%	2.38%
PTVBreastEval40G	P1	D 50.0% ≤ 43.20 Gy	41.50 Gy	39.84 Gy
PTVBreastEval40G	P1	V 48.00 Gy ≤ 30.0%	17.66%	12.66%
PTVBreastEval40G	P1	V 38.00 Gy ≥ 95.0%	95.09%	95.12%

**Table 5:** Comparison of dose metrics between VMAT and RAD for the lung case RAD, RapidArc Dynamic; VMAT, volumetric modulated arc therapy

Lung clinical goals				
Structure	Priority	Metric	VMAT	RAD
PTV-Plan	P1	D 2.0% < 107.0%	106.42%	105.16%
PTV-Plan	P1	D 5.0% < 105.0%	105.26%	103.80%
PTV-Plan	P1	D 95.0% > 95.0%	95.72%	95.10%
PTV-Plan	P1	D 99.0% > 90.0%	90.10%	91.62%
CORD	P1	Dmax < 44.00 Gy	42.64 Gy	41.35 Gy
HEART	P2	V 40.00 Gy < 20.0%	2.77%	2.64%
HEART	P3	V 5.00 Gy < 50.0%	60.75%	48.14%
HEART	P3	Dmean < 10.00 Gy	10.63 Gy	10.37 Gy
HEART	P3	V 30.00 Gy < 36.0%	6.51%	7.18%
LAD	P3	V 15.00 Gy < 10.0%	0.00%	0.00%
LUNG_LT	P2	V 5.00 Gy < 80.0%	12.04%	12.45%
Lungs-PTV	P2	V 20.00 Gy < 35.0%	24.99%	22.92%
Lungs-PTV	P2	Dmean < 18.00 Gy	15.04 Gy	14.70 Gy
OESOPHAGUS	P3	D 0.03 cm³ < 63.00 Gy	54.84 Gy	55.37 Gy
OESOPHAGUS	P3	Dmean < 34.00 Gy	16.34 Gy	16.23 Gy
OESOPHAGUS	P3	V 60.00 Gy < 10.0%	0.00%	0.00%
RT BRACH PLEXUS	P1	Dmax < 55.00 Gy	49.98 Gy	51.27 Gy

**Table 6:** Comparison of dose metrics between VMAT and RAD for the pancreas/liver case RAD, RapidArc Dynamic; VMAT, volumetric modulated arc therapy

Pancreas/liver clinical goals				
Structure	Priority	Metric	VMAT	RAD
PTV_40	P1	D 95.0% ≥ 38.00 Gy	40.29 Gy	40.81 Gy
PTV_40	P1	D 0.00 cm³ ≤ 52.00 Gy	45.34 Gy	46.18 Gy
PTV_50	P1	D 95.0% ≥ 47.50 Gy	50.84 Gy	50.97 Gy
PTV_50	P1	D 0.00 cm³ ≤ 65.00 Gy	56.48 Gy	57.82 Gy
Duodenum	P1	D 10.00 cm³ < 25.00 Gy	20.00 Gy	20.78 Gy
Duodenum	P1	D 0.50 cm³ < 35.00 Gy	28.80 Gy	29.50 Gy
Kidneys	P3	Dmean < 10.00 Gy	3.18 Gy	2.65 Gy
Liver-GTV	P2	Dmean ≤ 15.00 Gy	5.90 Gy	5.67 Gy
SmallBowel	P2	D 0.50 cm³ < 35.00 Gy	21.66 Gy	14.84 Gy
SmallBowel	P2	D 10.00 cm³ < 25.00 Gy	8.57 Gy	9.50 Gy
SpinalCord	P1	D 0.00 cm³ < 30.00 Gy	8.14 Gy	10.26 Gy
Stomach	P3	D 0.50 cm³ < 35.00 Gy	29.60 Gy	29.62 Gy
Stomach	P3	D 10.00 cm³ < 25.00 Gy	12.84 Gy	14.42 Gy

**Table 7:** Comparison of dose metrics between VMAT and RAD for the SBRT prostate case RAD, RapidArc Dynamic; SBRT, stereotactic body radiation therapy; VMAT, volumetric modulated arc therapy

SBRT prostate clinical goals				
Structure	Priority	Metric	VMAT	RAD
CTVp	P1	V 35.00 Gy ≥ 100.0%	97.81%	98.91%
GTVp	P1	V 50.00 Gy ≥ 100.0%	93.67%	99.60%
GTVp	P1	D 0.10 cm³ ≤ 52.00 Gy	51.80 Gy	52.18 Gy
PTV_P_P	P1	V 33.25 Gy ≥ 95.0%	94.34%	96.89%
Bladder_P1_P	P2	V 18.00 Gy ≤ 50.0%	8.97%	5.88%
Bladder_P1_P	P2	V 36.00 Gy ≤ 0.00 cm³	0.00 cm³	0.00 cm³
Bowel_P_P	P2	D 1.00 cm³ ≤ 0.00 Gy	1.23 Gy	1.22 Gy
Left Femoral He1	P3	Dmax ≤ 10.00 Gy	10.67 Gy	14.16 Gy
Neurovascular B1	P3	D 50.0% ≤ 35.00 Gy	35.48 Gy	35.35 Gy
penile bulb_P_P	P3	D 0.10 cm³ ≤ 10.00 Gy	1.64 Gy	1.46 Gy
Rectum_P1_P	P2	V 18.00 Gy ≤ 50.0%	26.30%	16.34%
Rectum_P1_P	P2	V 35.00 Gy ≤ 0.00 cm³	0.12 cm³	0.07 cm³
Rectum_P1_P	P2	D 40.0% ≤ 10.00 Gy	13.52 Gy	5.44 Gy
Right Femoral H1	P3	Dmax ≤ 10.00 Gy	13.28 Gy	13.02 Gy
Testes_P_P	P4	Dmax ≤ 0.00 Gy	0.29 Gy	0.30 Gy
Urethra_P_P	P2	D 20.0% ≤ 35.00 Gy	35.53 Gy	35.35 Gy

**Table 8:** Comparison of dose metrics between VMAT and RAD for the head and neck case RAD, RapidArc Dynamic; VMAT, volumetric modulated arc therapy

Head and neck clinical goals				
Structure	Priority	Metric	VMAT	RAD
PTV56	P1	V 56.00 Gy ≥ 97.0%	96.82%	96.80%
PTV63	P1	V 63.00 Gy ≥ 97.0%	95.07%	95.13%
PTV70	P1	V 70.00 Gy ≥ 97.0%	98.04%	96.85%
Brain	P3	V 50.00 Gy ≤ 100.00 cm³	0.97 cm³	0.02 cm³
Brain	P3	D 0.03 cm³ ≤ 70.00 Gy	60.85 Gy	47.95 Gy
Lips	P4	Dmean ≤ 20.00 Gy	23.63 Gy	22.74 Gy
Mandible-PTV	P4	V 50.00 Gy ≤ 31.0%	54.41%	56.92%
Mandible-PTV	P4	V 60.00 Gy ≤ 14.0%	18.89%	18.52%
Mandible-PTV	P4	V 70.00 Gy ≤ 6.5%	1.17%	0.30%
Ocavity-PTV	P4	Dmean ≤ 32.00 Gy	37.96 Gy	35.73 Gy
PharynxConst	P3	Dmean ≤ 25.00 Gy	47.82 Gy	46.51 Gy
SpinalCord_05	P2	V 30.00 Gy ≤ 30.0%	29.16%	15.88%
SpinalCord_05	P2	D 0.03 cm³ ≤ 45.00 Gy	45.89 Gy	42.67 Gy
SpinalCord_05	P2	V 40.00 Gy ≤ 5.0%	2.15%	0.32%

Breast case results

In Figure 2, the breast SIB case demonstrated significant dosimetric improvements with RAD. Most notably, RAD achieved a reduced heart mean dose (1.23 Gy vs. 1.98 Gy) and improved target dose homogeneity, with V42Gy reducing from 18.93% to 2.38%. The automatic skin flash management system contributed to better dose distribution while eliminating manual bolus planning complexity.

Lung case results

In Figure 3, the lung case, RAD yielded a substantial heart V5Gy reduction (48.1% vs. 60.8%), an important metric for reducing cardiovascular toxicity. This improvement was achieved through strategic STAMP placement and dynamic collimator rotation, optimizing heart avoidance.

Pancreas/liver case results

In Figure 4, the pancreas/liver case shows that the small bowel D0.5cc was reduced, demonstrating RAD’s ability to improve critical structure sparing even in complex multi-target scenarios.

Prostate case results

In Figure 5, the prostate plan demonstrated better sparing of both the bladder V18Gy (5.9% vs. 9.0%) and rectum V18Gy (16.3% vs. 26.3%). These improvements in critical structure sparing were achieved while maintaining equivalent target coverage.

Head and neck case results

In Figure 6, the head and neck case showed better sparing of sensitive organs such as the lips, oral cavity, and spinal cord. Specifically, the lips’ mean dose improved from 23.63 Gy to 22.74 Gy, and the oral cavity’s mean dose improved from 37.96 Gy to 35.73 Gy.

**Figure 7:**
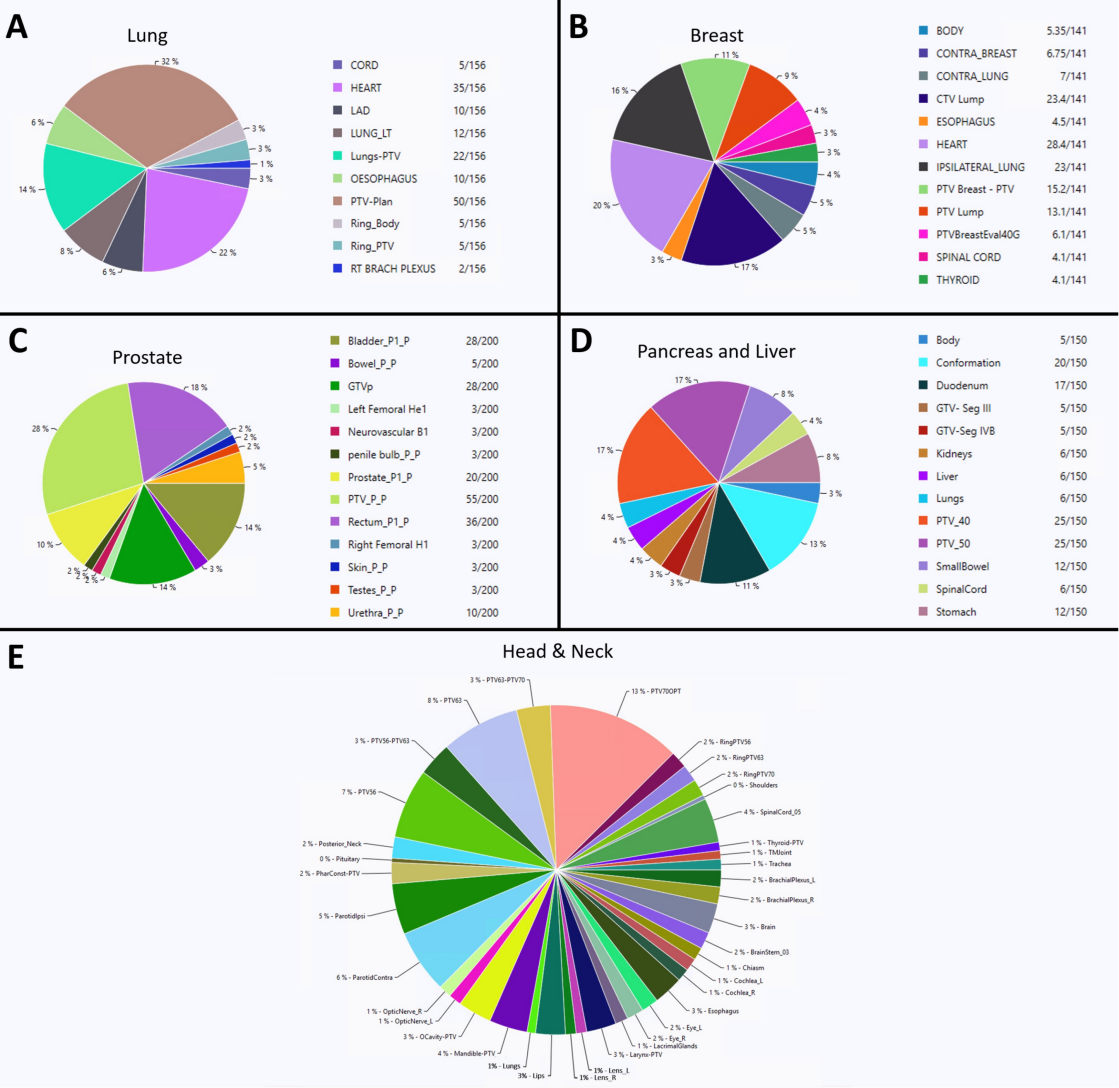
Pie charts showing the distribution of dosimetric scorecard points across the five treatment sites: (A) lung, (B) breast, (C) prostate, (D) pancreas/liver, and (E) head and neck This visualization helps identify the structures’ relative priority weighting for plan evaluation with each scorecard. Several structures have multiple metrics. The full scorecard for each case can be found online [13].

Dosimetric quality, as measured by each dosimetric scorecard, demonstrated improvements for all RAD plans (Appendix A, Appendix B, Appendix C, Appendix D, Appendix E). A visualization of how points are distributed between structures (relative weighting) is presented as pie charts in Figure 7. Optimization objectives to recreate these findings and per-metric results for each scorecard metric are available online [18]. Example scorecard piecewise linear functions for quantifying key metrics of improvement for RAD plans are shown in Figure 2C, Figure 3C, Figure 4C, Figure 5C, and Figure 6C. RAD had the same or improved beam on time in all but the pancreas/liver case.

## Discussion

Plan quality improvements

In the existing VMAT optimizer, high-quality treatment plans are possible. However, when more modulation is required, the user can only add arcs, even though modulation is likely to be needed at certain gantry rotation positions. This work demonstrates that treatment times can be reduced with similar or better dosimetric plan quality when those key gantry rotation positions are identified within the existing arc geometry and the system has the freedom to rotate the collimator for better results.

Breast case analysis

In the case of breast SIB, where most of the dose is to be delivered from tangent beam angles, STAMP selection is obvious. Dynamic collimator rotation throughout arc rotation allows jaws to constantly shield the heart and lungs (Video 1). In addition, RAD offers novel automatic integrated skin flash management, which varies the density of a virtual bolus relative to how tangent the gantry angle is positioned. Traditionally, manual virtual bolus techniques have been cumbersome to implement and result in unnecessarily heterogeneous dose distributions after final dose calculation on the patient’s true anatomy. Reduction in target dose heterogeneity (V105% <10%) was a key finding in reducing grade 3 dermatitis rates (<2%), as cited by Patel et al. [20]. These results align with Lin et al.’s findings that hybrid plans can achieve better heart sparing in breast cancer treatment while maintaining target coverage [6].

**Video 1:** Beam’s eye view of breast SIB RAD vs. VMAT RAD, RapidArc Dynamic; SIB, simultaneous integrated boost; VMAT, volumetric modulated arc therapy

Lung case analysis

The heart V5Gy reduction achieved in the lung case aligns with findings from RTOG 0617, which identified heart V5 as an independent prognostic factor for overall survival in stage III NSCLC patients [21]. This improvement highlights RAD’s enhanced dose modulation capabilities through dynamic collimator rotation and strategic STAMP placement.

Other treatment sites

The improvements seen in the head and neck case are consistent with previous findings from Akbas et al., who demonstrated enhanced target conformity and OAR sparing with hybrid techniques [5]. The enhanced sparing achieved across all treatment sites demonstrates RAD’s versatility in improving plan quality regardless of anatomical complexity.

Optimization efficiency analysis

These optimization efficiency improvements observed with RAD parallel those reported by Matuszak et al. with FusionArc [4]. Matuszak’s findings that “three beam FusionArc plans may offer a reasonable trade-off between final plan cost and MU/Gy” align with the observations of RAD’s efficiency, where a limited number of strategically placed STAMPs could significantly enhance plan quality without compromising delivery efficiency. This approach highlights the dosimetric quality that RAD can achieve in a shorter timeframe compared to VMAT in its faster, more clinically feasible settings.

Future directions

The impact of a faster RAD-type optimizer in the adaptive radiotherapy paradigm may make adaptive arc therapy plans more practical as well. The RAD cases in this study are available to download online to allow easy reuse of the beam geometry, STAMP weighting, and optimization objectives for testing and experimenting with this new technique [18].

Study considerations and limitations

The implementation of RAD represents a new paradigm in arc-based treatment plans, which offers noteworthy advantages and considerations. Unlike traditional VMAT optimization, RAD does not allow for interactively adjusting objectives and priorities. However, this potential limitation could be offset by quicker optimization times, facilitating a more trial-and-error-based approach where each trial time is minimal. The deterministic nature of RAD optimization ensures that identical objectives always produce identical results, eliminating the need for repeated optimization attempts due to variable convergence behavior and further enhancing planning efficiency. Potential overall planning time could be reduced or similar, depending on the number of trials a planner attempts. Anecdotally, RAD’s deterministic approach has shown that treatment planners are often willing to explore aggressive optimization objectives. The fast and complete reproducibility of each optimization eliminates concern about compromising the progress made during longer interactive optimizations, encouraging more thorough exploration of the solution space.

A current challenge in implementing RAD is determining the optimal configuration of STAMPs (their number, location, and weighting), which requires customization for different disease sites and anatomical locations. While this process can be time-consuming initially, it offers the potential for highly tailored and efficient treatment plans. Many treatment sites are amenable to a class solution, such as the breast, prostate, and head and neck cases in this study. For these cases, readers are invited to try the specific STAMPs and weighting examples demonstrated in this manuscript and provided online [18]. Whereas, with the lung and pancreas/liver cases, specific STAMPs and weighting were selected based on each patient’s specific anatomy. As such, these STAMPs are less likely to be useful for other patients.

Another limitation of this study was that the optimization objectives were originally tuned for VMAT, transferred to RAD, further tuned for best results, and then copied back to VMAT (Table 9). Objectives were not tuned a final time for the VMAT results. This limitation could have potentially left unrealized dosimetric plan quality improvements for the VMAT plans in this comparison. Ultimately, using the same RAD objectives for the VMAT plans seemed to be the fairest way of evaluating the new RAD features against VMAT.

**Table 9:** Comparison of plan quality scores and MU across the three experimental phases for five different treatment sites Groups include an initial VMAT plan, a RAD plan with further objective tuning, and a final re-optimized VMAT plan that utilized identical RAD objectives. MU, monitor unit; RAD, RapidArc Dynamic; VMAT, volumetric modulated arc therapy

Plan quality score timeline			
Plan type	Initial VMAT	RAD	Final VMAT
Breast plan score	102.49	128.4	104.93
Breast plan MU	817	1036	842
Lung plan score	94.58	107.26	95.45
Lung plan MU	665	742	721
Pancreas/liver plan score	132.81	139.66	134.16
Pancreas/liver plan MU	3625	3630	3535
Prostate plan score	156.88	178.02	167.87
Prostate plan MU	2326	3602	2231
Head and neck plan score	179.15	196.45	181.45
Head and neck plan MU	824	1182	924

In addition, all attempts were made to find the best possible plans without time constraints. Due to the allowed unlimited planning time, all improvements demonstrated in this work may not be realized in routine clinical practice. Cases planned and delivered in a real-world clinical planning setting will help determine any practical clinical advantage.

The final limitation of this study was the inability to perform delivery verification measurements. Verification pass rates and dosimetric accuracy analysis would provide crucial validation of RAD plan deliverability and should be included in future studies. While the delivery time measurements confirmed successful plan execution, comprehensive quality assurance measurements, including gamma analysis, would be required before any clinical implementation.

## Conclusions

This study demonstrates that RAD can offer advantages in radiation therapy planning and delivery. RAD notably reduces optimization time compared to traditional VMAT. Importantly, plan quality is either maintained or improved with RAD, indicating no compromise in treatment effectiveness despite faster planning. The potential for reduced delivery times with RAD could further enhance treatment efficiency and patient experience. These findings suggest that RAD represents an advancement in radiation therapy, potentially allowing for more efficient clinical workflows without sacrificing plan quality. DICOM, optimization objectives, clinical goals, and dosimetric scorecards for the RAD cases shown in this work are available for download online.

## Appendices

Appendix A 

**Table 10:** Comparison of dose metrics between VMAT and RAD for the SIB breast case RAD, RapidArc Dynamic; SIB, simultaneous integrated boost; VMAT, volumetric modulated arc therapy

SIB breast scorecard comparison							
Structure	Metric	Goal	VMAT value	RAD value	VMAT score	RAD score	Possible points per metric
CTV Lump	Dose at 98%	≥ 48.0 Gy	49.67 Gy	47.96 Gy	13.2	12.98	13.2
CTV Lump	Dose at 2CC	≤ 48.67 Gy	52.37 Gy	49.64 Gy	0	4.44	5.1
CTV Lump	Dose at 0.1CC	≤ 49.77 Gy	53.33 Gy	50.58 Gy	0	4.26	5.1
PTV Lump	Dose at 95%	≥ 46.32 Gy	47.44 Gy	45.95 Gy	13.1	11.73	13.1
HEART	Mean Dose	≤ 1.2 Gy	1.98 Gy	1.23 Gy	8.7	9.95	10.1
HEART	Dose at 2CC	≤ 5.45 Gy	23.44 Gy	19.06 Gy	0.62	2.38	8.1
HEART	Dose at 10%	≤ 2.0 Gy	2.98 Gy	2.01 Gy	2.6	4.98	5.1
HEART	Dose at 5%	≤ 2.5 Gy	4.16 Gy	2.79 Gy	3.77	4.8	5.1
IPSILATERAL_LUNG	Mean Dose	≤ 5.0 Gy	4.28 Gy	3.12 Gy	6.24	6.31	6.5
IPSILATERAL_LUNG	Volume at 4Gy	≤ 38.0%	29.13%	18.04%	6.54	6.9	7
IPSILATERAL_LUNG	Volume at 12Gy	≤ 25.0%	7.41%	4.38%	4.85	4.91	5
IPSILATERAL_LUNG	Volume at 18Gy	≤ 14.0%	3.70%	2.50%	4.43	4.46	4.5
PTVBreastEval40G	Dose at 90%	≥ 38.77 Gy	39.15 Gy	38.58 Gy	6.1	5.97	6.1
PTV Breast – PTV	Dose at 90%	≥ 38.77 Gy	38.61 Gy	38.33 Gy	7.94	7.84	8.1
PTV Breast – PTV	Volume at 42.05Gy	≤ 5.0%	18.20%	2.19%	0.85	6.73	7.1
CONTRA_LUNG	Mean Dose	≤ 1.28 Gy	0.66 Gy	0.22 Gy	3.24	3.41	3.5
CONTRA_LUNG	Volume at 2.5Gy	≤ 10.0%	1.01%	0.00%	3.47	3.5	3.5
CONTRA_BREAST	Mean Dose	≤ 1.6 Gy	0.61 Gy	0.15 Gy	3.15	3.23	3.25
CONTRA_BREAST	Dose at 2CC	≤ 5.0 Gy	1.74 Gy	0.57 Gy	3.41	3.47	3.5
ESOPHAGUS	Volume at 24Gy	≤ 5.0%	0.00%	0.00%	4.5	4.5	4.5
SPINAL CORD	Dose at 0.04CC	≤ 7.0 Gy	1.91 Gy	1.37 Gy	4.1	4.1	4.1
THYROID	Volume at 24Gy	≤ 2.0%	0.00%	0.00%	4.1	4.1	4.1
BODY	Max Dose	≤ 50.4 Gy	54.25 Gy	51.14 Gy	0	3.47	5.35
Total score					104.93	128.4	141

Appendix B

**Table 11:** Comparison of dose metrics between VMAT and RAD for the lung case RAD, RapidArc Dynamic; VMAT, volumetric modulated arc therapy

Lung scorecard comparison							
Structure	Metric	Goal	VMAT value	RAD value	VMAT score	RAD score	Possible points per metric
PTV-Plan	Volume at 95%	≥ 95.0%	96.07%	95.25%	12.86	10.66	21
PTV-Plan	Dose at 95%	≥ 95.0%	95.72%	95.10%	2.52	0.35	10
PTV-Plan	Dose at 99%	≥ 90.0%	90.10%	91.62%	0.1	1.62	5
PTV-Plan	Dose at 2%	≤ 105.0%	106.43%	105.16%	1.42	4.6	8
PTV-Plan	Dose at 5%	≤ 103.0%	105.27%	103.80%	0	2.4	6
Lungs-PTV	Volume at 20Gy	≤ 30.0%	25.00%	22.93%	12	12.64	14
LUNG_LT	Volume at 5Gy	≤ 50.0%	12.04%	12.45%	12	12	12
Lungs-PTV	Mean Dose	≤ 15.0 Gy	15.04 Gy	14.70 Gy	3.95	4.3	8
LAD	Volume at 15Gy	≤ 5.0%	0.00%	0.00%	10	10	10
CORD	Dose at 0.03CC	≤ 44.0 Gy	41.75 Gy	40.50 Gy	2.25	3.5	5
HEART	Dose at 0.03CC	≤ 69.0 Gy	63.80 Gy	62.83 Gy	5	5	5
HEART	Mean Dose	≤ 10.0 Gy	10.63 Gy	10.37 Gy	10.92	11.37	15
HEART	Volume at 30Gy	≤ 30.0%	6.52%	7.19%	10	10	10
HEART	Volume at 5Gy	≤ 60.0%	60.75%	48.14%	2.78	4.59	5
OESOPHAGUS	Dose at 0.1CC	≤ 55.0 Gy	53.68 Gy	54.36 Gy	1.06	0.51	5
OESOPHAGUS	Mean Dose	≤ 37.0 Gy	16.34 Gy	16.23 Gy	5	5	5
RT BRACH PLEXUS	Dose at 0.03CC	≤ 55.0 Gy	45.78 Gy	49.62 Gy	2	2	2
Ring_PTV	Dose at 0.03CC	≤ 63.0 Gy	62.80 Gy	60.64 Gy	0.3	3.54	5
Ring_Body	Dose at 0.03CC	≤ 60.0 Gy	57.98 Gy	55.06 Gy	1.3	3.17	5
Total Score					95.45	107.26	156

Appendix C 

**Table 12:** Comparison of dose metrics between VMAT and RAD for the pancreas/liver case RAD, RapidArc Dynamic; VMAT, volumetric modulated arc therapy

Pancreas/liver scorecard comparison							
Structure	Metric	Goal	VMAT value	RAD value	VMAT score	RAD score	Max score
PTV_50	Volume at 50Gy [%]	≥ 98.0%	98.54%	99.61%	18.54	19.61	20
PTV_40	Volume at 40Gy [%]	≥ 98.0%	97.02%	99.52%	15.4	19.52	20
GTV- Seg III	Dose at 0.03CC [Gy]	≥ 40.0 Gy	40.36 Gy	40.26 Gy	5	5	5
GTV-Seg IVB	Dose at 0.03CC [Gy]	≥ 40.0 Gy	40.30 Gy	40.92 Gy	5	5	5
PTV_40	Dose at 0.03CC [Gy]	≥ 39.6 Gy	38.49 Gy	39.30 Gy	3.27	4.53	5
PTV_50	Dose at 0.03CC [Gy]	≥ 49.5 Gy	48.89 Gy	49.82 Gy	4.24	5	5
Conformation	Conformation No. at 40Gy	≥ 0.6	0.68	0.65	8.78	8.53	10
Conformation	Conformation No. at 20Gy	≥ 0.15	0.14	0.14	6.36	5.08	10
Body	Mod GI [V20/V40] Gy	≤ 6.5	4.91	4.76	5	5	5
Duodenum	Dose at 0.03CC [Gy]	≤ 34.0 Gy	34.45 Gy	34.24 Gy	6.68	6.83	7
Duodenum	Volume at 20Gy [CC]	≤ 10.0 CC	10.02 CC	12.17 CC	5	4.57	5
Duodenum	Volume at 30Gy [CC]	≤ 1.0 CC	0.31 CC	0.41 CC	5	5	5
SmallBowel	Volume at 20Gy [CC]	≤ 0.5 CC	0.82 CC	0.01 CC	3.91	4	4
SmallBowel	Volume at 30Gy [CC]	≤ 0.0 CC	0.0004 CC	0.0000002 CC	4	4	4
SmallBowel	Volume at 35Gy [CC]	≤ 0.0 CC	0.00 CC	0.0000002 CC	4	4	4
Stomach	Volume at 20Gy [CC]	≤ 5.0 CC	3.09 CC	3.70 CC	3	3	3
Stomach	Volume at 30Gy [CC]	≤ 0.0 CC	0.45 CC	0.46 CC	2.66	2.66	3
Stomach	Volume at 35Gy [CC]	≤ 0.0 CC	0.10 CC	0.08 CC	2.8	2.84	3
Stomach	Volume at 40Gy [CC]	≤ 0.0 CC	0.00 CC	0.00 CC	3	3	3
SpinalCord	Dose at 0.03CC [Gy]	≤ 7.0 Gy	7.70 Gy	9.69 Gy	2.9	2.62	3
SpinalCord	Dose at 0.35CC [Gy]	≤ 10.0 Gy	6.79 Gy	8.50 Gy	3	3	3
Kidneys	Dose at 200CC [Gy]	≤ 0.0 Gy	3.10 Gy	2.73 Gy	2.54	2.59	3
Kidneys	Volume at 23Gy [%]	≤ 0.0%	0.00%	0.00%	3	3	3
Liver	Dose at 700CC [Gy]	≤ 2.0 Gy	5.09 Gy	4.39 Gy	2.38	2.52	3
Liver	Mean Dose [Gy]	≤ 5.0 Gy	6.07 Gy	5.84 Gy	2.75	2.81	3
Lungs	Dose at 1500CC [Gy]	≤ 0.0 Gy	0.18 Gy	0.18 Gy	2.96	2.96	3
Lungs	Volume at 13.5Gy [%]	≤ 0.0%	0.00%	0.00%	3	3	3
Total Score					134.16	139.66	150

Appendix D

**Table 13:** Comparison of dose metrics between VMAT and RAD for the prostate case RAD, RapidArc Dynamic; SBRT, stereotactic body radiation therapy; VMAT, volumetric modulated arc therapy

SBRT prostate scorecard comparison							
Structure	Metric	Goal	VMAT value	RAD value	VMAT score	RAD score	Possible points per metric
Prostate_P1_P	Volume at 35Gy [%]	≥ 95.0%	97.83%	98.85%	19.13	19.54	20
PTV_P_P	Volume at 33.25Gy [%]	≥ 95.0%	94.61%	96.95%	34.02	35	35
GTVp	Volume at 50Gy [%]	≥ 97.0%	93.66%	99.60%	17.38	19.74	20
GTVp	Dose at 0.1CC [Gy]	≤ 52.0 Gy	51.81 Gy	52.18 Gy	8	7.28	8
PTV_P_P	Dose at 100% [Gy]	≥ 31.8 Gy	29.09 Gy	30.76 Gy	8.36	9.37	10
PTV_P_P	Conformation No. at 31.8Gy	≥ 0.6	0.86	0.84	6.46	6.04	10
Rectum_P1_P	Volume at 31.5Gy [CC]	≤ 1.0 CC	1.34 CC	1.28 CC	8.89	9.66	15
Rectum_P1_P	Dose at 40% [Gy]	≤ 8.75 Gy	13.53 Gy	5.45 Gy	9.23	13	13
Rectum_P1_P	Volume at 18Gy [CC]	≤ 12.5 CC	14.85 CC	9.22 CC	7.62	8	8
Bladder_P1_P	Volume at 18Gy [CC]	≤ 12.5 CC	34.84 CC	22.85 CC	3.64	6.34	8
Bladder_P1_P	Volume at 32.4Gy [CC]	≤ 3.0 CC	2.22 CC	1.82 CC	10.55	11.36	15
Bladder_P1_P	Volume at 36Gy [CC]	≤ 5.0 CC	0.00 CC	0.00 CC	5	5	5
Urethra_P_P	Dose at 20% [Gy]	≤ 35.0 Gy	35.53 Gy	35.35 Gy	8.49	9	10
Bowel_P_P	Dose at 1CC [Gy]	≤ 26.3 Gy	1.23 Gy	1.22 Gy	4.77	4.77	5
penile bulb_P_P	Dose at 0.1CC [Gy]	≤ 10.0 Gy	1.64 Gy	1.47 Gy	3	3	3
Neurovascular B1	Dose at 50% [Gy]	≤ 35.0 Gy	35.49 Gy	35.35 Gy	2.41	2.58	3
Right Femoral H1	Dose at 0.03CC [Gy]	≤ 8.75 Gy	12.90 Gy	12.88 Gy	2.55	2.55	3
Left Femoral He1	Dose at 0.03CC [Gy]	≤ 8.75 Gy	10.37 Gy	13.50 Gy	2.86	2.41	3
Skin_P_P	Dose at 0.03CC [Gy]	≤ 8.75 Gy	7.38 Gy	21.08 Gy	3	0.89	3
Testes_P_P	Dose at 0.03CC [Gy]	≤ 1.75 Gy	0.29 Gy	0.30 Gy	2.5	2.49	3
Total Score					167.87	178.02	220

Appendix E 

**Table 14:** Comparison of dose metrics between VMAT and RAD for the head and neck case (sample of total metrics due to higher number) RAD, RapidArc Dynamic; SBRT, stereotactic body radiation therapy; VMAT, volumetric modulated arc therapy

Head and neck scorecard comparison (subsample)							
Structure	Metric	Goal	VMAT value	RAD value	VMAT score	RAD score	Possible points per metric
PTV56	Volume at 56Gy [%]	≥ 95.0%	96.82%	96.80%	14.37	14.35	15
PTV56	Dose at 99% [Gy]	≥ 53.2 Gy	53.71 Gy	54.80 Gy	0.93	1.23	1.5
PTV63	Volume at 63Gy [%]	≥ 95.0%	95.07%	95.13%	13.11	13.19	17
PTV63	Dose at 99% [Gy]	≥ 59.85 Gy	59.19 Gy	60.47 Gy	0	0.95	1.5
PTV70	Volume at 70Gy [%]	≥ 95.0%	98.04%	96.84%	19.35	18.69	20
PTV70	Dose at 99% [Gy]	≥ 67.2 Gy	69.49 Gy	69.32 Gy	1.41	1.38	1.5
PTV70	Dose at 0.03CC [Gy]	≤ 77.0 Gy	76.02 Gy	74.96 Gy	7.7	8.46	10
Brain	Dose at 0.03CC [Gy]	≤ 70.0 Gy	60.78 Gy	47.85 Gy	1.58	1.7	2
Brain	Volume at 10Gy [%]	≤ 5.0%	6.89%	4.04%	2.49	4.19	5
Lips	Mean Dose [Gy]	≤ 15.0 Gy	23.63 Gy	22.74 Gy	3.19	3.63	7
Mandible-PTV	Volume at 70Gy [%]	≤ 6.5%	1.17%	0.30%	4.73	4.93	5
Mandible-PTV	Volume at 60Gy [%]	≤ 14.0%	18.89%	18.52%	1.34	1.37	2
Mandible-PTV	Volume at 50Gy [%]	≤ 31.0%	54.41%	56.92%	0.39	0.26	2
OCavity-PTV	Mean Dose [Gy]	≤ 30.0 Gy	37.96 Gy	35.73 Gy	2.96	3.43	6
OCavity-PTV	Dose at 0.03CC [Gy]	≤ 60.0 Gy	60.82 Gy	63.28 Gy	1.64	1.32	2
PharynxConst	Mean Dose [Gy]	≤ 25.0 Gy	17.38 Gy	16.70 Gy	4.75	4.78	5
SpinalCord_05	Dose at 0.03CC [Gy]	≤ 50.0 Gy	45.87 Gy	42.64 Gy	5.65	6.06	6.5
SpinalCord_05	Volume at 40Gy [%]	≤ 10.0%	2.16%	0.32%	1.78	1.97	2
SpinalCord_05	Volume at 30Gy [%]	≤ 45.0%	29.23%	15.92%	1.51	1.73	2
Total Score					88.88	93.62	113
